# Study on detection of pesticide residues in tobacco based on hyperspectral imaging technology

**DOI:** 10.3389/fpls.2024.1459886

**Published:** 2024-09-30

**Authors:** Min Liang, Zhiqiang Wang, Yu Lin, Caixia Li, Liang Zhang, Yaxi Liu

**Affiliations:** ^1^ Triticeae Research Institute, Sichuan Agricultural University, Chengdu, Sichuan, China; ^2^ College of Information Engineering, Sichuan Agricultural University, Ya ‘an, China; ^3^ Institute of Agronomy and Horticulture, Chengdu Agricultural College, Chengdu, Sichuan, China

**Keywords:** pesticide residue detection, hyperspectral imaging technology, machine learning, support sector machine, food safety

## Abstract

**Introduction:**

Tobacco is a critical economic crop, yet its cultivation heavily relies on chemical pesticides, posing health risks to consumers, therefore, monitoring pesticide residues in tobacco is conducive to ensuring food safety. However, most current research on pesticide residue detection in tobacco relies on traditional chemical methods, which cannot meet the requirements for real-time and rapid detection.

**Methods:**

This study introduces an advanced method that combines hyperspectral imaging (HSI) technology with machine learning algorithms. Firstly, a hyperspectral imager was used to obtain spectral data of tobacco samples, and a variety of spectral pre-processing technologies such as mean centralization (MC), trend correction (TC), and wavelet transform (WT), as well as feature extraction methods such as competitive adaptive reweighted sampling (CARS) and least angle regression (LAR) were used to process the spectral data, and then, grid search algorithm (GSA) is used to optimize the support sector machine (SVM).

**Results:**

The optimized MC-LAR-SVM model achieved a pesticide classification accuracy of 84.1%, which was 9.5% higher than the original data model. The accuracy of the WT-TC-CARS-GSA-SVM model in the fenvalerate concentration classification experiment was as high as 91.8 %, and it also had excellent performance in other metrics. Compared with the model based on the original data, the accuracy, precision, recall, and F1-score are improved by 8.3 %, 8.2 %, 7.5 %, and 0.08, respectively.

**Discussion:**

The results show that combining spectral preprocessing and feature extraction algorithms with machine learning models can significantly enhance the performance of pesticide residue detection models and provide robust, efficient, and accurate solutions for food safety monitoring. This study provides a new technical means for the detection of pesticide residues in tobacco, which is of great significance for improving the efficiency and accuracy of food safety detection.

## 1 Introduction

Tobacco is one of the most important economic crops in the world, necessitating the employment of chemical control methods in its cultivation to minimize the adverse effects of pests and diseases on both its yield and quality (Lecours et al., 2012). However, the use of chemical pesticides can have adverse effects on the environment and the health of smokers (Dai et al., 2019). Therefore, the issue of pesticide residue has always been a major concern within the tobacco industry.

Traditional pesticide residue detection techniques include Gas Chromatography (GC), Gas Chromatography-Mass Spectrometry (GC-MS), High-Performance Liquid Chromatography (HPLC), Supercritical Fluid Chromatography (SFC) and other methods, although these detection methods have high accuracy for the detection of pesticide residues, the detection process is relatively complicated and costs labor and financial resources (Grimalt and Dehouck, 2016; Zhang et al., 2018; Pérez-Navarro et al., 2019; Simonetti et al., 2020).

Hyperspectral imaging (HSI) is a technique that combines spectral technology with image technology to obtain spectral information in each pixel space of the research object (Gowen et al., 2007). In recent years, HSI technology has garnered significant attention in the field of pesticide residue detection. Ye et al. (2022) established an LR model using HSI technology to effectively detect pesticide residue levels in grapes, with an accuracy of more than 97%. He et al. (2021) combined HSI technology to establish a one-dimensional convolutional neural networks (1D-CNN) model to detect pesticide residues in leek leaves and the accuracy of the test set was 97.9%. Jun et al. (2016) utilized HSI technology and chlorophyll fluorescence spectroscopy to quantitatively analyze the residues of dichlorvos at various concentrations on lettuce leaves and the SVM model exhibited a strong correlation coefficient of 0.987 and a low root mean square error of 0.005. Jia et al. (2018) developed a detection model for cypermethrin and carbendazim pesticide residues on apple surfaces by integrating HSI technology with a machine learning algorithm, achieving a classification accuracy of 95%. In conclusion, HSI technology has been widely utilized for non-destructive detection of pesticide residues in agricultural products. However, most current research on pesticide residue detection in tobacco relies on traditional chemical detection methods, which cannot meet the requirements for real-time and rapid detection. Hyperspectral imaging technology offers the advantages of rapid, non-destructive, and accurate quality detection and has been extensively applied across various crops. Therefore, it is feasible to employ hyperspectral imaging technology for detecting the levels of pesticide residues on the surface of tobacco leaves.

As high-dimensional data, including spectral and spatial features, it is still a difficult task to mine the effective information of hyperspectral data. The utilization of machine learning algorithms is crucial in the prediction and analysis of spectral information, as it enables automatic pattern recognition and rule derivation from data, leading to accurate predictions. Its advantages lie in its automation, adaptability, high efficiency, and predictive capabilities, making it widely applicable in pesticide residue detection (Saha and Manickavasagan, 2021). Tan et al. (2024) utilized HSI technology for the detection of pesticide residues in cantaloupe, achieving an SVM model with an accuracy of up to 93.13%. Zhan-qi et al. (2018) utilized HSI technology in conjunction with a machine learning algorithm to detect dimethoate pesticide residues at various concentrations in spinach leaves. The optimal linear discrimination model achieved a prediction accuracy of 0.997 with a standard deviation of 0.008. Cong et al. (2021) developed a support vector machine model utilizing HSI technology for the identification of pesticide residues on lettuce surfaces, achieving a test set accuracy of 96.08%. Sun et al. (2022) utilized fluorescence spectroscopy technology to develop a random forest classifier model based on a one-dimensional convolutional neural network for the detection of pesticide residues on black tea, achieving a test set accuracy of 99.05%.

In this study, HSI technology was utilized to analyze various types of pesticides and their residues to capture spectral discrepancies among different pesticide types or residues. Concurrently, a machine learning algorithm detection model was developed and fine-tuned.

## 2 Materials and methods

### 2.1 Sample handling

Fenthion, dimethoate, and fenvalerate, which were purchased from Merck with a purity greater than 98%, were used as test agents. Tobacco plants were cultivated in the greenhouse of Sichuan Agricultural University with a photosynthetic photon flux density (PPFD) of 600 μmol·m^-2^·s^-1^ and a photoperiod of 16 h light (25°C), and 8 h dark (22°C) for four weeks.

After dilution at a ratio of 1:1000, the test agent was uniformly sprayed onto the surface of tobacco leaves. Distilled water was used as the control. Each group consisted of 20 tobacco plants, designated as T_1_ - T_4_, each plant selected 3-5 leaves for hyperspectral imaging, details of the sample are shown in **Table 1**.

**Table 1:** Sample processing of different pesticide classification experiments.

Group name	Category	Dilution ratio	Number of tobacco (plant)	Sample size
T_1_	fenthion	1:1000	20	412
T_2_	Dimethoate	1:1000	20	382
T_3_	Fenvalerate	1:1000	20	447
T_4_	water	–	20	429

Fenvalerate was diluted at ratios of 1:200, 1:1000, 1:500, 1:25,000, and 1:100,000 before being evenly sprayed onto the surface of tobacco leaves. Distilled water was used as the control. Each group consisted of 20 tobacco plants labeled C_1_ – C_6_, each plant selected 3-5 leaves for hyperspectral imaging, details of the sample are shown in **Table 2**.

**Table 2:** Sample treatment of fenvalerate concentration grading experiment.

Group name	Dilution ratio	Number of tobacco (plant)	Sample size
C_1_	1:200	20	68
C_2_	1:1000	20	78
C_3_	1:5000	20	67
C_4_	1:25000	20	68
C_5_	1:100000	20	81
C_6_	–	20	68

### 2.2 Hyperspectral image acquisition and correction

**Figure 1:**
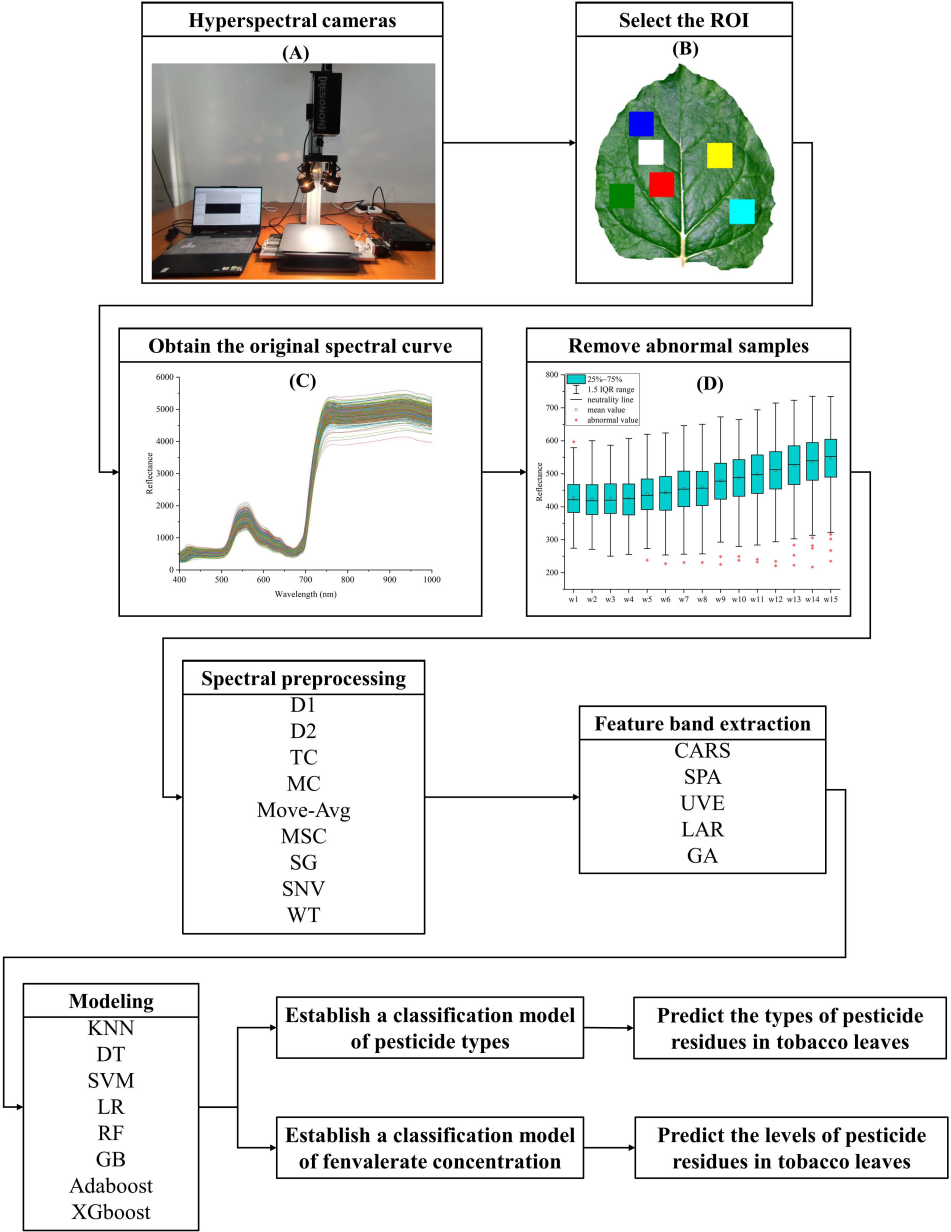
Workflow of hyperspectral data acquisition, processing and modeling. **(A)** Hyperspectral imager. **(B)** Selection of ROI. **(C)** Original spectral curve of fresh tobacco leaf sample. **(D)** Box plot method to remove abnormal samples.

In this study, Resonon’s Pika XC2 hyperspectral imager was used for spectral image acquisition. It covers the spectral range from 400 nm to 1000 nm with a spectral resolution of 1.9 nm, encompassing 462 bands, more details are shown in **Table 3**. As shown in **Figure 1A**, the entire system comprises the imaging spectrometer (Pika XC2), camera light sources (The host model is DMX-J-SA-17), stage, and computer.

**Table 3:** Hyperspectral cameras specifications.

Hyperspectral cameras specifications	Pika XC2
Spectral range (nm)	400-1000
Spectral resolution (nm)	1.9
Bin spectral channels	447
Spatial channels	1600
Max frame rate (fps)	165
Sampling interval (nm)	1.3
Weight (kg)	2.51
Connection type	USB
Dimensions (cm)	26.5 x10.6 x 7.5

Each tobacco leaf sample was positioned horizontally on the stage for data acquisition. The original hyperspectral images were then grayscale corrected to reduce noise caused by changes in illumination and camera dark current. This was achieved by placing a standard reflection whiteboard perpendicular to the imaging lens and capturing a single frame of whiteboard data corresponding to the current slit was acquired. Furthermore, by employing a lens cover over the lens, corresponding black frame data could be obtained. The calculation is as follows:


(1) $$I=\frac{R-B}{W-B}\times 100\%$$

W is all-white image reflectivity; B is all-black image reflectance; R is the reflectance of the original spectral image; I is the reflectance of the corrected spectral image.

### 2.3 Data analysis

#### 2.3.1 Hyperspectral data extraction

As shown in **Figure 1B**, The ENVI5.6 software was used to select the 80 × 80pixel region at the location to avoid the vein as the region of interest (ROI), and the average spectral value within ROI was calculated as the original spectrum of the sample (**Figure 1C**).

#### 2.3.2 Spectral preprocessing and sample division

In the process of spectral data acquisition, different environmental factors and individual differences of samples may introduce interference such as stray light, strong electric noise, and artificial transmission noise, which will affect the establishment of the model and the final detection result. The application of the spectral preprocessing algorithm has been proven to significantly improve classification accuracy (Mishra et al., 2020).

**Figure 2:**
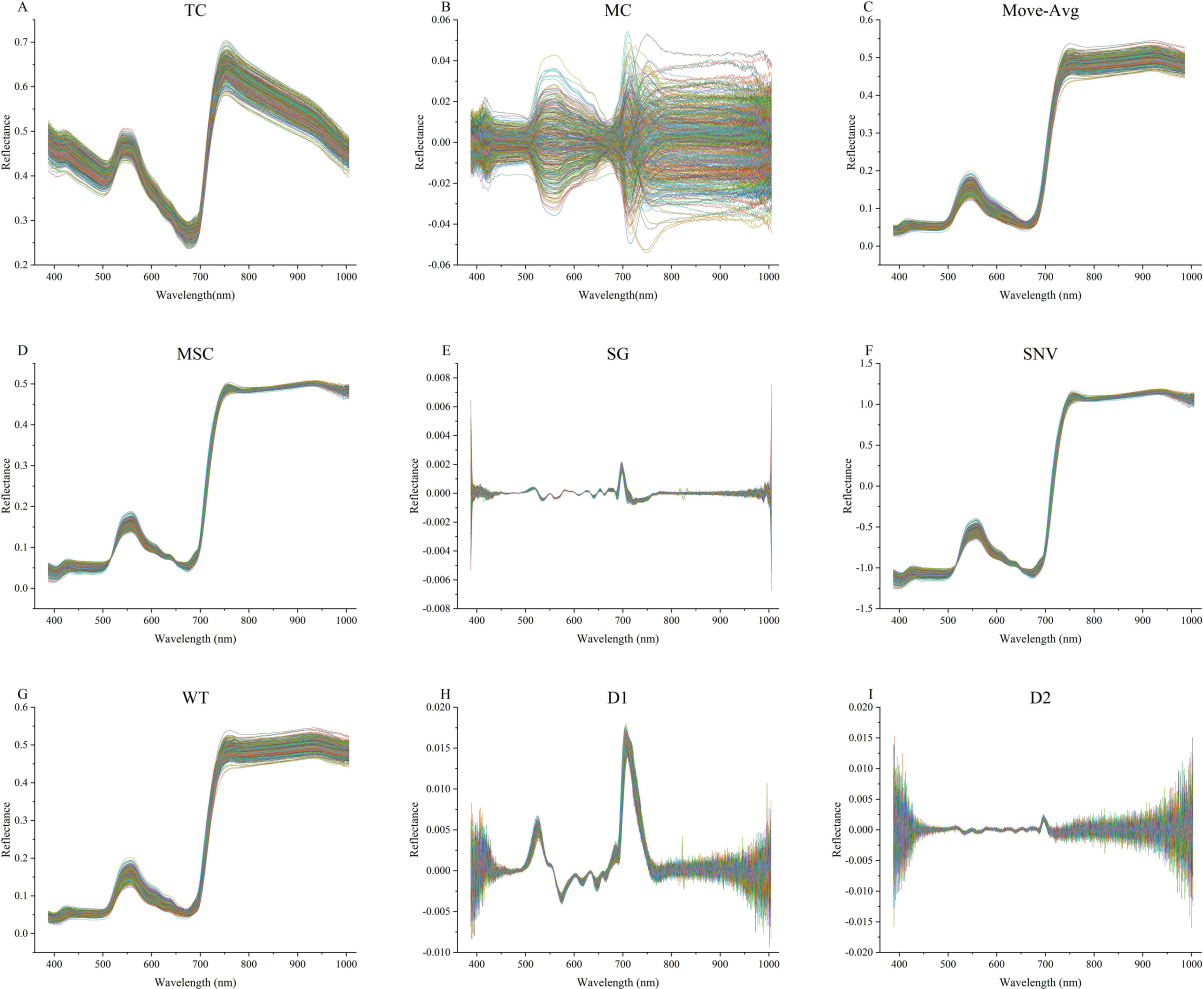
The spectral curve after pretreatment. **(A)** Spectra after trend correction. **(B)** Spectra after mean centering. **(C)** Spectra after moving average filters. **(D)** Spectra after multiple scattering correction. **(E)** Spectra after savitzky-golay. **(F)** Spectra after standard normalized variable. **(G)** Spectra after wavelet transform. **(H)** Spectra after first order differential. **(I)** Spectra after second order differences.

As shown in **Figure 1D**, after the box plot method is used to eliminate abnormal samples, first order differential (D1), second order differences (D2), trend correction (TC), mean centering (MC), moving average filters (Move-Avg), multiple scattering correction (MSC), savitzky-golay (SG) smoothing, standard normalized variable (SNV), wavelet transform (WT) are respectively used for spectral data preprocessing (**Figures 2A-F, H, I**). In this study, the specific parameters are set as follows: in the Move-Avg algorithm, the window size is specified as 2. In the SG algorithm, the window size is specified as 21 and the polynomial order is 3 to smooth the data, to reduce the random fluctuations in the data. In WT algorithm, Daubechies 8 wavelet is used to transform, and the noise is suppressed by setting the threshold value to 0.04. Use the default settings for other algorithms. The results of modeling based on nine methods were compared and analyzed, and the best spectral pretreatment algorithm was selected for subsequent research and analysis. The preprocessed spectral data set is randomly divided into a training set and a test set according to the ratio of 4:1 for model training.

#### 2.3.3 Spectral feature extraction method

To improve the accuracy and efficiency of hyperspectral data modeling, a feature selection algorithm is employed to address redundant information and dimensionality issues (Medjahed and Ouali, 2018). In this study, we used the competitive adaptive reweighted sampling (CARS), successive projection algorithm (SPA), uninformative variable elimination (UVE), least angle regression (LAR), and genetic algorithm (GA) for feature band extraction, the specific parameters are set as follows: In the CARS algorithm, 50 subsets, 20 maximum principal component numbers, and 10 cross-validations are used to determine the optimal wavelength. In the SPA algorithm, variable selection is done by automatic scaling and default minimum variable number 1. The UVE algorithm evaluates features by setting the number of potential components to 50, the number of repetitions to 500, and the size of the test set to 20%. GA algorithm sets the crossover probability as 0.5 and the mutation probability as 0.2, and sets the variable selection threshold as 0.5 to determine the selected characteristic variable. The LAR algorithm uses default Settings for feature selection. The modeling results based on five methods were compared and analyzed, and the best feature band extraction algorithm was selected for subsequent research and analysis.

#### 2.3.4 Software environment

The training was performed on a local machine with an NVIDIA GeForce RTX 4060 GPU and a 13th Gen Intel(R) Core (TM) i713700F CPU. Use Python language, Keras 2.4.3, scikit-learn 0.24.1, and Tensorflow 2.4.1 served as the framework, with CUDA 11.8 and cuDNN 8.9.4 for acceleration.

#### 2.3.5 Establishment and evaluation of tobacco pesticide residue detection model

In this study, k-nearest neighbor (KNN), decision tree (DT), support vector machine (SVM), logistic regression (LR), random forest (RF), gradient boosting (GB), adaboost and extreme gradient boosting (XGboost) were chosen to construct models for pesticide residue detection. To ensure a fair and comprehensive comparison of the performance of various machine learning algorithms for pesticide residue detection, all models were initially trained using their default parameters. This approach was taken to establish a baseline for model performance without any bias introduced by parameter tuning.

After establishing the baseline performance, the grid search algorithm (GSA) was then applied to fine-tune the models. GSA systematically explores a predefined range of parameter values to identify the combination that maximizes model performance, as evaluated by the chosen metrics: accuracy (A), precision (P), recall (R), and F1-score (F). These metrics are shown in Equation 2, Equation 3, Equation 4, and Formula 5.


(2) $$Accuracy=\frac{TP+TN}{TP+TN+FP+FN}$$

(3) $$Precision=\frac{TP}{TP+FP}$$

(4) $$Recall=\frac{TP}{TP+FN}$$

(5) $$F1-score=\frac{2*Recall*Precision}{Recall+Precision}$$

TP is to predict the correct category as the correct category. FN is to predict the correct category as the wrong category. FP is to predict the wrong category as the correct category. TN is to predict the wrong category as the wrong category.

**Figure 1** summarizes the workflow from sample collection to data modeling.

## 3 Results and analysis

### 3.1 Extraction and analysis of spectral information

The extracted spectral data is shown in **Figure 1C**. Within the 400 ~ 700nm range, a minor peak in reflectivity emerges around the 560nm green light, while a trough in reflectivity appears near the 700nm red light. There is a sharp increase in reflectance in the 700 ~ 780nm range. Within the band of 780 ~ 1000nm, reflectivity tends to stabilize.

**Figure 3:**
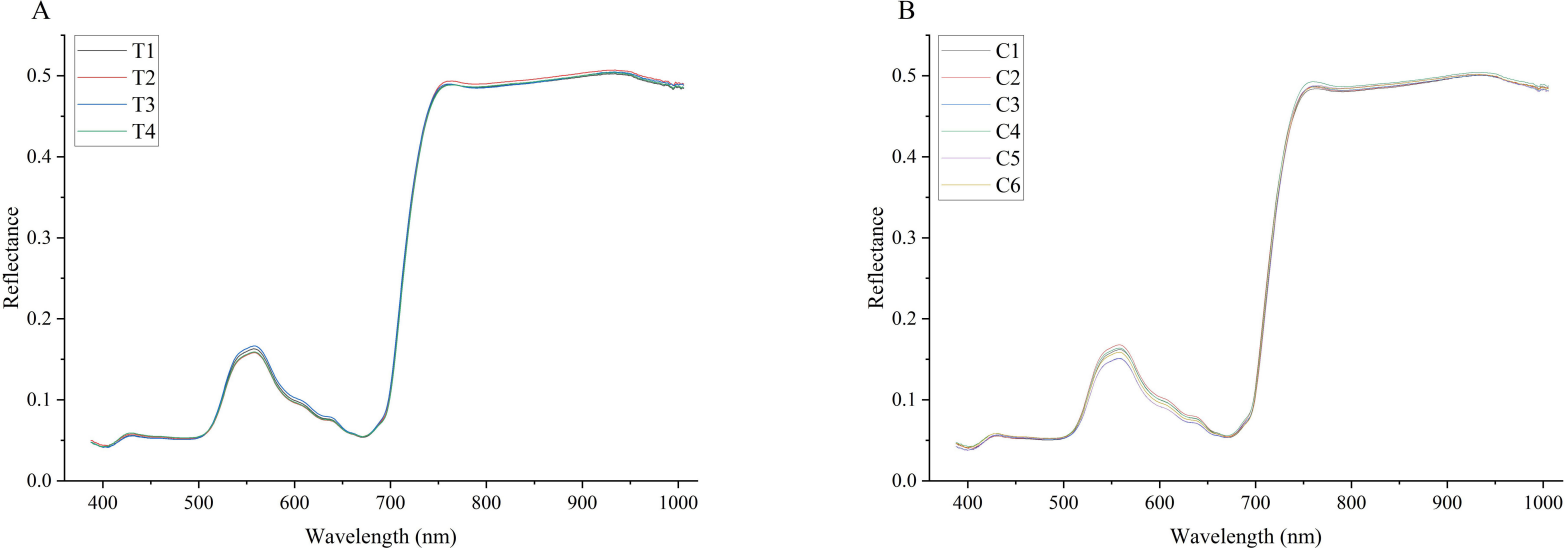
**(A)** The average spectral curve of the three pesticides, in which T_1_, T_2_, and T_3_ are fenthion, dimethoate, and fenvalerate, and T_4_ is treated with water as the control. **(B)** the average spectral curve of six concentration levels of fenvalerate, in which C_1-5_ were diluted according to 1:200, 1:1000, 1:500, 1:25,000, and 1:100,000 respectively, and C_6_ was treated with water as the control.

To clearly discern the differences in spectral curves among tobacco samples within each group, the average spectral curves for each type of sample were computed as depicted in **Figure 3**, where T_1_ – T_4_ represents the average spectral curves for various pesticide species. C_1_ – C_6_ denotes the average spectrum of fenvalerate diluted in varying proportions.

The spectral curves in **Figure 3** demonstrate that the average trends of all samples are largely similar, with peaks and troughs occurring at approximately 560, 700, and 800nm. However, these features do not overlap completely. At these specific wavelengths, the spectral reflectance of tobacco leaves varies depending on the type and concentration of pesticide applied. This suggests that it is feasible to identify pesticide residues in tobacco and potentially quantify their concentrations using spectral information.

### 3.2 Spectral preprocessing

**Figure 4:**
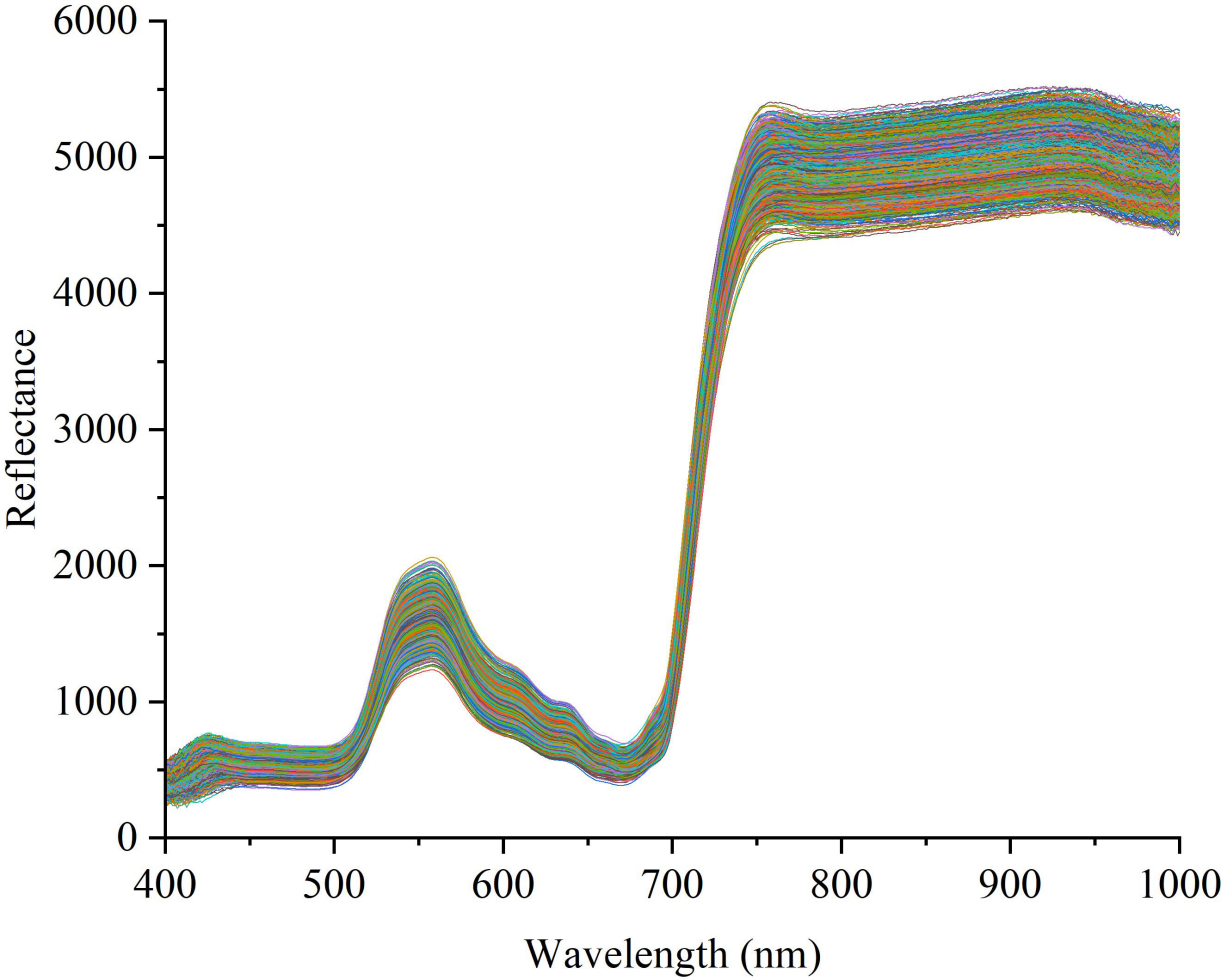
Spectral curve of fresh tobacco leaf samples after removal of outliers.

The spectral curve after the removal of abnormal samples is shown in **Figure 4**. The box plot method can effectively remove outliers in the data according to the distribution characteristics of the data. In this study, we used 9 kinds of spectral preprocessing techniques to process the spectral data and conducted a comprehensive evaluation of their effects. As shown in **Figure 2**, the original spectral data underwent different pretreatment methods, and its characteristic performance and noise level changed significantly. For example, D1 and D2 reduce the impact of baseline fluctuations by highlighting spectral features, but this may also be responsible for the loss of some important spectral information, as reflected in the lower performance indicators in **Table 4**. TC and MC effectively remove the uncorrelated variability in the spectrum and improve the prediction accuracy of the model, which is reflected in the high accuracy, precision, recall, and F1-score in **Table 4**. In particular, the MC method performs well in the classification of pesticide residues, the identification model of different pesticide species established using spectral data processed by MC outperforms the original model by 8% in accuracy, 7.5% in precision, 8.2% in recall, and 0.08 in F1-score.

**Table 4:** Classification results of nine preprocessing algorithms.

Preprocessing	Classification of pesticide types				Concentration classification of fenvalerate			
	A (%)	P (%)	R (%)	F	A (%)	P (%)	R (%)	F
Raw	74.6	74.5	73.3	0.73	83.5	83.5	84.2	0.83
D1	76.0	75.8	74.8	0.75	74.1	73.0	72.2	0.72
D2	68.3	67.5	66.5	0.66	64.7	62.7	62.5	0.61
TC	78.4	78.5	77.3	0.77	87.1	87.3	87.7	0.86
MC	82.6	82.0	81.5	0.81	83.5	83.5	84.2	0.83
Move-Avg	71.0	70.8	70.0	0.70	80.0	80.7	79.3	0.78
MSC	75.7	75.3	74.3	0.74	83.5	82.7	82.7	0.82
SG	77.5	76.5	76.3	0.76	67.1	67.3	67.0	0.66
SNV	48.2	48.8	48.3	0.48	48.2	51.3	47.5	0.45
WT	76.6	75.5	75.3	0.75	84.7	85.0	85.3	0.84
WT-TC	74.0	73.0	72.5	0.72	89.4	89.2	89.0	0.88

While the purpose of using SNV was to reduce the effects due to changes in the physical properties of the sample, it resulted in a substantial decline in performance in our dataset, possibly due to its excessive standardization process which weakened useful spectral information. As shown in **Figure 2G**, WT improved the performance of the model by removing high-frequency noise components, especially in the fenvalerate concentration classification experiment, its F1-score reached 0.84. However, when WT is combined with TC (WT-TC), although the performance in the classification experiment of pesticide residues is decreased, the highest accuracy of 89.4%, precision of 89.2%, recall of 89.0%, and F1-score of 0.88, are achieved in the fenvalerate concentration classification, indicating that the combination of the two methods can provide the best prediction effect under certain circumstances.

### 3.3 Extraction of spectral feature bands

Raw spectral data (Raw) is used as a benchmark, and its performance indicator reflects the classification ability of the model without the application of a feature band extraction algorithm. As shown in **Table 5**, the original data achieved an accuracy of 74.6% in the classification of pesticide types and 83.5% in the concentration classification of fenvalerate. These baseline results provide a reference for subsequent comparisons.

**Table 5:** Classification results based on five feature band extraction algorithms.

Feature band extraction	Classification of pesticide types				Concentration classification of fenvalerate			
	A (%)	P (%)	R (%)	F	A (%)	P (%)	R (%)	F
Raw	74.6	74.5	73.3	0.73	83.5	83.5	84.2	0.83
CARS	72.5	72.5	71.8	0.72	81.2	80.0	79.2	0.79
SPA	26.6	6.8	25.0	0.10	74.1	74.0	73.5	0.73
UVE	74.9	74.3	73.8	0.73	84.7	84.5	84.2	0.83
LAR	78.7	78.5	77.8	0.77	61.2	60.5	59.2	0.59
GA	76.3	76.8	75.5	0.75	83.5	83.5	83.8	0.82

The performance of the CARS method in pesticide classification was slightly lower than the original data, and the accuracy dropped to 72.5%, indicating that CARS may not effectively extract out the optimal characteristic bands in this dataset. The performance of CARS also decreased in concentration classification. The performance of SPA in the classification of pesticide types was significantly lower than other methods, with an accuracy of only 26.6%, which may indicate that SPA had limitations in processing the spectral data of this study and did not retain enough information for the model to accurately classify. The UVE method showed a similar performance to the original data in the classification of pesticide species, with an accuracy of 74.9%, while the fenvalerate concentration classification showed a similar performance to the original data, with an accuracy of 84.7%, indicating that UVE maintained the classification ability of the model in feature selection. LAR performed well in the classification of pesticide species with an accuracy of 78.7%, the highest of all methods, but it performed poorly in the concentration classification of fenvalerate with an accuracy of only 61.2%, which may mean that the characteristic bands selected by LAR are not representative enough for the concentration classification task. GA showed good performance in both classification tasks, the accuracy of pesticide classification was 76.3%, while the accuracy of fenvalerate concentration classification was 83.5%, which was close to the performance of the original data, indicating that GA had high robustness in the selection of characteristic bands (**Table 5**).

### 3.4 Classification model building

**Figure 5:**
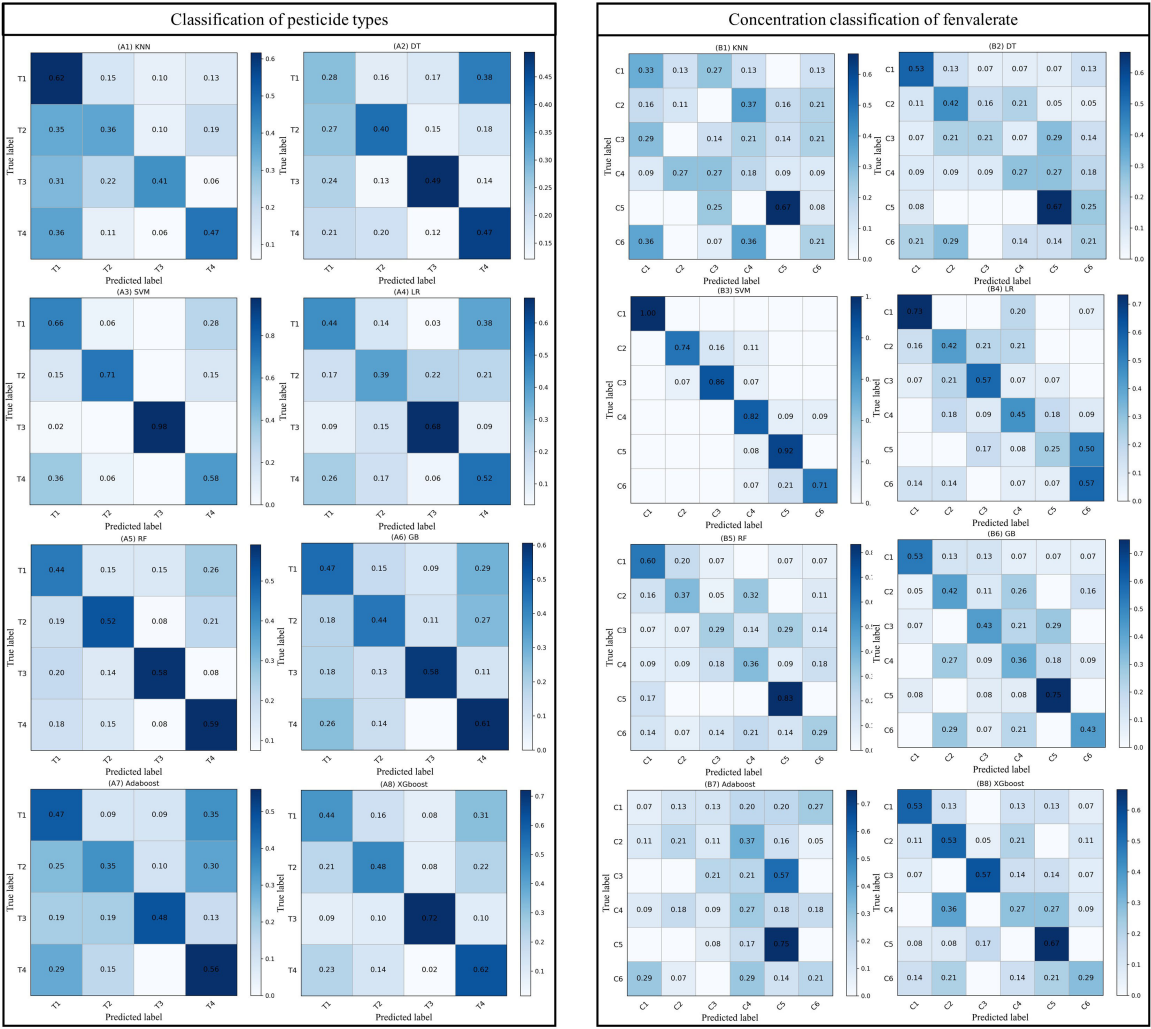
Confusion matrix for different machine learning models, in which T_1_, T_2_, and T_3_ are fenthion, dimethoate, and fenvalerate, and T_4_ is treated with water as the control. C_1-5_ indicated that fenvalerate was diluted according to 1:200, 1:1000, 1:500, 1:25,000, and 1:100,000 respectively, and C_6_ was treated with water as the control.

In this study, Firstly, we comprehensively evaluated the performance of eight machine learning algorithms on pesticide classification and fenvalerate concentration classification tasks. By analyzing the confusion matrix and the classification results in **Table 6**, the results showed that SVM showed excellent classification accuracy in all the evaluated categories, reaching 74.6% in the classification task of pesticide types, and the F1-score was 0.73. The fenvalerate concentration classification task was far ahead with an accuracy of 83.5% and an F1-score of 0.83, underscoring the SVM’s ability to handle high-dimensional data and nonlinear problems. As shown in **Figure 5**, RF and GB show balanced performance in most categories, but the diagonal values are not optimal in some specific categories, suggesting possible overfitting or feature space mismatches. Although LR algorithm performed slightly better than KNN and DT in pesticide classification, it did not show obvious advantages in fenvalerate concentration classification task. Relatively speaking, KNN and DT have poor classification performance on multiple categories, which may be due to their limitations when dealing with complex data distributions. Adaboost and XGboost, while performing well in some categories, are inconsistent overall. To sum up, this study selects SVM as the benchmark model for experimental analysis.

**Table 6:** Classification results of eight machine learning algorithms.

Model	Classification of pesticide types				Concentration classification of fenvalerate			
	A (%)	P (%)	R (%)	F	A (%)	P (%)	R (%)	F
KNN	46.1	48.5	46.5	0.46	25.9	26.8	27.3	0.26
DT	41.0	41.5	41.0	0.41	37.3	38.5	38.5	0.37
SVM	74.6	74.5	73.3	0.73	83.5	83.5	84.2	0.83
LR	50.9	51.0	50.8	0.50	50.6	49.5	49.8	0.49
RF	53.0	53.3	53.3	0.53	44.7	43.8	45.7	0.43
GB	51.8	53.5	52.5	0.51	48.2	50.2	48.7	0.48
Adaboost	45.8	48.5	46.5	0.46	27.1	27.7	28.7	0.25
XGboost	56.6	57.0	56.5	0.56	48.2	48.5	47.7	0.47

Secondly, we thoroughly analyzed the effects of combining different spectral pretreatment techniques and characteristic wavelength extraction algorithms on the performance of pesticide residue classification models. According to the results in **Table 7**, it can be clearly seen that the specific combination of pretreatment and feature selection significantly improves the classification effect of the model. In particular, after selecting the features of MC combined with LAR, SVM was adopted as the classifier (MC-LAR-SVM), which performed well in the classification experiment of pesticide types. The average classification accuracy of the test set reached 84.1%, the precision was 83.3%, the recall was 83.0%, and the F1-score was 0.83. Compared with the SVM model based on raw data, the average classification accuracy increased by 9.5%, the precision increased by 8.8%, the recall increased by 9.7%, and the F1-score increased by 0.1. Additionally, the training time of the MC-LAR-SVM model is 52.18 seconds, which is not the shortest compared with other combination methods, but considering its advantages in classification performance, this training time is acceptable. This result highlights the effectiveness of MC and LAR in enhancing spectral data characteristics and reducing noise, thereby improving the predictive power of models.

**Table 7:** The results of pesticide classification experiment.

Preprocessing	Feature band extraction	A (%)	P (%)	R (%)	F	Training time (second)
–	–	74.6	74.5	73.3	0.73	12.84
D1	CARS	74.9	74.0	74.0	0.74	21.41
	SPA	28.7	28.8	28.8	0.28	4.03
	UVE	67.7	67.3	66.5	0.66	24.73
	LAR	75.7	75.5	73.5	0.74	0.55
	GA	61.7	61.5	60.8	0.60	68.18
D2	CARS	61.7	61.3	61.3	0.61	94.00
	SPA	34.4	34.3	34.3	0.34	2.52
	UVE	66.8	66.8	66.3	0.66	120.18
	LAR	68.9	68.3	67.5	0.67	1.46
	GA	59.0	58.3	57.8	0.57	331.74
TC	CARS	76.6	76.0	75.8	0.75	4.78
	SPA	32.0	16.0	30.5	0.19	0.22
	UVE	73.7	73.3	73.0	0.72	4.55
	LAR	75.7	76.5	74.8	0.75	10.05
	GA	71.6	71.3	70.3	0.70	7.78
MC	CARS	79.9	79.3	79.0	0.79	480.24
	SPA	46.4	44.3	45.0	0.44	0.54
	UVE	77.8	76.8	77.0	0.76	690.23
	LAR	84.1	83.3	83.0	0.83	52.18
	GA	76.0	76.0	75.3	0.75	1393.16
Move-Avg	CARS	72.8	72.5	72.0	0.72	3.44
	SPA	34.1	18.0	31.0	0.21	0.22
	UVE	68.6	69.5	68.0	0.68	2.49
	LAR	72.8	73.0	72.0	0.72	6.48
	GA	71.2	71.3	70.5	0.70	6.66
MSC	CARS	79.0	78.5	78.0	0.78	1.02
	SPA	25.7	6.5	25.0	0.10	0.22
	UVE	73.4	72.8	72.8	0.72	0.58
	LAR	75.4	75.3	74.5	0.74	1.60
	GA	72.5	72.5	71.8	0.71	3.23
SG	CARS	73.4	73.0	73.0	0.72	0.48
	SPA	38.9	38.5	38.5	0.37	4.80
	UVE	76.9	76.3	76.3	0.76	2.49
	LAR	77.5	76.5	76.3	0.73	1.53
	GA	69.5	68.8	68.8	0.68	2.92
SNV	CARS	45.5	46.0	46.0	0.44	0.06
	SPA	37.7	35.8	36.5	0.29	1.67
	UVE	36.8	29.3	37.8	0.31	0.03
	LAR	48.2	48.8	48.3	0.48	0.14
	GA	44.9	45.5	45.3	0.43	0.10
WT	CARS	76.0	75.5	75.8	0.75	5.10
	SPA	27.8	7.0	25.0	0.11	0.22
	UVE	66.8	67.5	65.8	0.66	3.88
	LAR	74.3	73.3	73.0	0.73	9.79
	GA	71.9	70.8	70.8	0.70	6.06

Then, we conducted a comprehensive analysis of fenvalerate concentration classification. As shown in **Table 8**, among the various combinations, the WT-TC-CARS-SVM model has the best performance, reaching an average classification accuracy of 90.6%, and also has an excellent performance in precision, recall, and F1-score, which are 90.3%, 90.3% and 0.9 respectively. It is worth noting that the WT-TC-CARS-SVM model not only has excellent classification performance but also shows good efficiency in training time (1.27 seconds), which indicates the potential and advantages of this model in practical applications.

**Table 8:** The results of concentration classification experiment of fenvalerate.

Preprocessing	Feature band extraction	A (%)	P (%)	R (%)	F	Training time (second)
–	–	83.5	83.5	84.2	0.83	1.00
D1	CARS	67.1	67.0	65.7	0.65	0.02
	SPA	50.6	50.5	49.5	0.48	0.50
	UVE	70.6	69.0	68.0	0.68	0.00
	LAR	55.3	54.3	55.0	0.54	0.03
	GA	70.1	70.0	68.7	0.68	0.02
D2	CARS	48.2	50.8	47.8	0.47	0.03
	SPA	27.1	26.7	26.3	0.24	0.06
	UVE	52.9	51.7	52.0	0.50	0.02
	LAR	44.7	43.2	44.0	0.42	0.63
	GA	62.4	62.5	61.0	0.60	0.02
TC	CARS	87.1	86.7	86.2	0.86	79.0
	SPA	57.6	53.8	54.3	0.53	0.69
	UVE	84.7	85.2	84.2	0.83	0.56
	LAR	69.4	69.3	69.0	0.68	1.12
	GA	87.1	88.8	85.8	0.86	0.63
MC	CARS	83.5	83.8	81.7	0.82	2.90
	SPA	76.5	77.2	77.2	0.76	12.41
	UVE	85.9	85.0	85.3	0.85	0.41
	LAR	63.5	63.0	62.2	0.62	26.18
	GA	89.4	89.7	90.0	0.89	0.29
Move-Avg	CARS	71.8	72.3	71.5	0.71	2.25
	SPA	72.9	72.8	73.3	0.72	1.30
	UVE	75.3	75.0	75.0	0.74	3.37
	LAR	74.1	75.2	74.5	0.74	3.18
	GA	76.5	75.0	75.3	0.74	1.77
MSC	CARS	84.7	85.8	83.8	0.84	0.09
	SPA	56.5	55.5	56.8	0.55	0.31
	UVE	85.9	84.7	84.7	0.84	0.08
	LAR	56.5	58.3	56.8	0.56	0.10
	GA	76.5	75.2	74.8	0.74	0.14
SG	CARS	75.3	74.2	74.3	0.74	0.01
	SPA	45.9	43.3	43.5	0.43	0.02
	UVE	69.4	69.3	69.2	0.68	0.00
	LAR	57.6	58.2	57.2	0.55	0.00
	GA	63.5	63.5	63.7	0.61	0.05
SNV	CARS	36.5	26.5	36.2	0.28	0.02
	SPA	14.1	2.3	16.7	0.4	0.02
	UVE	38.8	51.7	38.2	0.32	0.02
	LAR	27.1	10.2	25.8	0.13	0.00
	GA	37.6	51.2	37.3	0.31	0.00
WT	CARS	83.5	84.2	82.7	0.82	1.13
	SPA	64.7	64.2	63.7	0.63	1.16
	UVE	80.0	78.8	79.3	0.78	0.49
	LAR	56.5	55.7	57.2	0.55	2.87
	GA	84.7	85.0	85.2	0.84	0.58
WT-TC	CARS	90.6	90.3	90.3	0.90	1.27
	SPA	72.9	72.2	72.2	0.71	1.41
	UVE	87.1	86.5	86.2	0.85	0.60
	LAR	84.7	83.8	84.2	0.83	0.41
	GA	81.2	80.7	81.2	0.80	0.91

Finally, we used the fenvalerate concentration classification experiment as an example to optimize the model parameters by grid search algorithm (GSA). The results in **Table 9** show that after parameter optimization, the recognition rate has been improved to some extent. Specifically, the Raw-SVM model has demonstrated a high accuracy of 83.5% without any preprocessing. The accuracy of the WT-TC-SVM model was improved to 89.4%. Furthermore, the average classification accuracy of the WT-TC-CARS-GSA-SVM model on the test set reached 91.8%. This significant performance improvement is not only reflected in the accuracy but also the precision, recall, and F1-score. Compared with the Raw-SVM model, the accuracy, precision, recall, and F1-score were improved by 8.3%, 8.2%, 7.5%, and 0.08, respectively. After GSA parameter tuning, it is determined that the kernel function is a multinomial kernel function (Poly), the best penalty factor c is 0.1, and the best kernel parameter g is 1e-4.

**Table 9:** The results of fenvalerate concentration classification ablation experiment.

Model	A (%)	P (%)	R (%)	F
Raw-SVM	83.5	83.5	84.2	0.83
WT-TC-SVM	89.4	89.2	89.0	0.88
CARS-SVM	81.2	80.0	79.2	0.79
WT-TC-CARS-SVM	90.6	90.3	90.3	0.90
WT-TC-CARS-GSA-SVM	91.8	91.7	91.7	0.91

## 4 Discussion

The hyperspectral data of tobacco collected in this study showed “green peak”, “red valley”, “red edge” and “infrared high step” (**Figure 1C**), which aligns with the observed trend in spectral curves discussed by predecessors, indicating conformity with the spectral reflectance properties of green leaves (Asner, 1998; Ma et al., 2023).

To enhance the signal-to-noise ratio by reducing spectral noise, this study used nine preprocessing methods for spectral data. Notably, the combined WT-TC processing method further optimized model performance. Compared with the model based on the original data, the accuracy rate increased from 83.5% to 89.4%. Furthermore, compared with the research conducted by Sun et al. (2022) on the detection of pesticide residues in black tea, the accuracy of the MSC-SVM and SNV-SVM models was improved from 81% to 84.8% and 82.9%, respectively, whereas the model established based on the WT-TC combined pretreatment method in this study achieved even higher accuracy. The results show that the detection performance of the model can be significantly improved by selecting the preprocessing technology suitable for the data characteristics.

Although full-band spectral data can be utilized for modeling, the redundant information within the data may lead to decreased computational efficiency and recognition accuracy of the model (Lei et al., 2022). In the two experiments of this study, it can be found that when the pre-processing or feature extraction algorithm is only applied to the spectral data, the model performance improvement space is small, and sometimes even the model performance is reduced. For example, in the fenvalerate experiment, the accuracy of the established CARS-SVM model was even reduced by 2.3% compared with Raw-SVM, while the accuracy of the WT-TC-CARS-SVM model established by CARS combined with WT-TC was increased by 7.1%. Similar research results have been reported in previous studies. Sun et al. (2023) utilized HSI technology to investigate pesticide residues on lettuce leaves and achieved correlation coefficients of 0.8132 for the full-band model and 0.8234 for the feature-band model they established. Furthermore, when applying a convolutional smoothing pre-processing algorithm in combination with principal component analysis for feature extraction, the predictive correlation coefficient of their established model reached 0.901, representing a significant improvement over the feature band-based model. In addition, to explore how the model works, this study attempted to draw a decision boundary graph to better evaluate the model. For this reason, principal component analysis was used to reduce the dimensionality of the data. Unfortunately, when we reduced the data of the 462 band to only two dimensions, it resulted in a considerable loss of key features, and the classification result of the established model was quite poor, with the accuracy reduced to 28.1%. In summary, choosing the right feature band extraction algorithm, combined with effective preprocessing techniques, is critical to optimizing the performance of the model to reach its maximum potential.

In comparison with eight machine learning models, the SVM model demonstrated superior performance and higher classification accuracy on the test set, whether in the context of pesticide residue identification or fenvalerate concentration classification experiments. SVM also demonstrated superior performance and higher predictive accuracy compared to other machine learning algorithms (Jia et al., 2018; Hu et al., 2023). This can be attributed to SVM’s ability to select an optimal decision boundary by maximizing the margin and its capacity to map data into a higher-dimensional feature space using kernel functions, thereby exhibiting strong generalization capabilities and enabling nonlinear classification (Kotsiantis et al., 2006).

The partial least squares (PLS) model established by Shen et al. (2022), achieved an average classification accuracy of 80% for the test set of three pesticides in detecting pesticide residues in cauliflower, while the MC-LAR-SVM model developed in this study demonstrated a recognition accuracy of 84.1%, showing a significant improvement of 4.1%. Compared with the study of pesticide residue detection in beef by Jiang et al. (2022), the accuracy of multilayer perceptron (MLP), SVM, and RF models was 88.6%, 87.6%, and 86.2% respectively, while the WT-TC-CARS-GSA-SVM model developed in this study for fenvalerate concentration classification reached an accuracy of 91.8%. In a word, the optimization of spectral pretreatment technology and feature extraction algorithm highlights its great potential in improving the detection accuracy of pesticide residues and provides strong technical support for the strengthening of food safety monitoring.

## 5 Conclusion

The study successfully explored the possibility and efficiency of combining hyperspectral imaging technology with machine learning algorithms for pesticide residue detection. Through a series of experiments, this study verified the effectiveness of the proposed method in the task of pesticide types classification and fenvalerate concentration classification. The SVM model, especially the SVM model after WT-TC-CARS pretreatment and GSA parameter optimization performed well in improving classification accuracy (WT-TC-CARS- GSA-SVM), with an average classification accuracy of 91.8% on the test set, and the precision, recall, and F1-score were 90.3%, 90.3% and 0.9, respectively, which outperforms the original model by 8.3% in accuracy, 8.2% in precision, 7.5% in recall, and 0.08 in F1-score. Furthermore, the enhanced model maintains a comparable level of computational complexity to that of the original model. The experimental results confirm the effectiveness of the comprehensive pretreatment and feature extraction algorithm in improving the model performance, which is of great significance in realizing rapid and accurate pesticide residue analysis.

However, the study also revealed the limitations of this approach. The influence of environmental factors on hyperspectral data acquisition, equipment cost, computational cost and time of model training, and the limitation of model generalization ability are all problems that need to be further solved in the practical application of this technology.

Future research should focus on improving the stability of data collection, lowering the technical threshold, enhancing the generalization and interpretation ability of the model, and developing more efficient optimization algorithms to promote the practical application of this technology in the field of pesticide residue detection.

In conclusion, this study provides a new technical approach for the rapid detection of pesticide residues in tobacco and provides empirical support for the combined application of hyperspectral imaging technology and machine learning algorithms. As the technology continues to advance and optimize, we believe that this method will have the potential to play a greater role in the field of food safety detection.

## Data Availability

The original contributions presented in the study are included in the article/supplementary material. Further inquiries can be directed to the corresponding authors.
